# Comprehensive Review of Urinary Tract Infections in Renal Transplant Recipients: Clinical Insights and Management Strategies

**DOI:** 10.7759/cureus.53882

**Published:** 2024-02-08

**Authors:** Vidhi Bharuka, Revat Meshram, Pratiksha K Munjewar

**Affiliations:** 1 Pediatrics, Jawaharlal Nehru Medical College, Datta Meghe Institute of Higher Education and Research, Wardha, IND; 2 Medical Surgical Nursing, Smt. Radhikabai Meghe Memorial College of Nursing, Datta Meghe Institute of Higher Education and Research, Wardha, IND

**Keywords:** multidisciplinary care, prevention strategies, antibiotic resistance, immunosuppression, renal transplant recipients, urinary tract infections

## Abstract

Urinary tract infections (UTIs) pose a significant challenge in the care of renal transplant recipients. This comprehensive review explores this population's multifaceted landscape of UTIs, emphasizing the importance of early diagnosis and tailored management strategies. Renal transplant recipients face an elevated risk of UTIs due to immunosuppression, altered urinary tract anatomy, and complex comorbidities. Complications of UTIs can lead to graft dysfunction and systemic illness, underscoring the need for effective management. The emergence of multidrug-resistant uropathogens adds complexity to treatment, highlighting the importance of targeted antibiotic therapy. Antibiotics are the most commonly prescribed drugs for UTIs, with nitrofurantoin, fosfomycin, amoxicillin, and amoxicillin-clavulanate potassium being some of the commonly used antibiotics. However, the emergence of multidrug-resistant uropathogens has led to the exploration of alternative treatments, such as bacteriophage therapy, as a potential alternative against multidrug-resistant uropathogenic bacteria. Analgesics such as phenazopyridine can be prescribed to relieve discomfort associated with UTIs. Estrogen therapy has also been suggested as a potential treatment option for UTIs, particularly in postmenopausal women. Trimethoprim-sulfamethoxazole or trimethoprim is recommended as first-line therapy for uncomplicated UTIs. The choice of drug and therapy for UTIs depends on the severity of the infection, the causative organism, and the presence of antibiotic resistance. Preventive measures encompass pre-transplant evaluation, perioperative strategies, post-transplant follow-up, and vaccination. A multidisciplinary approach involving transplant specialists, infectious disease experts, pharmacists, and patient engagement is vital for successful care. The future of UTI management lies in ongoing research, exploring personalized medicine, novel therapies, and innovative prevention strategies. By implementing these strategies and advancing research, healthcare providers can improve graft and patient survival, enhancing the quality of care for renal transplant recipients.

## Introduction and background

Urinary tract infections (UTIs) are a well-recognized and common complication in renal transplant recipients. These infections encompass a range of microbial invasions affecting various parts of the urinary system, including the bladder, urethra, and, more critically, the transplanted kidney itself. UTIs hold unique clinical significance in renal transplantation due to the complex interplay of immunosuppressive regimens, altered urinary tract anatomy, and the lifelong implications for graft function and patient well-being [1]. UTIs in renal transplant recipients are distinctive in their potential to disrupt the delicate balance between immune suppression and graft protection. Unlike in the general population, where UTIs are often self-limiting, the consequences can be dire in this vulnerable patient group. The significance lies in potential complications, ranging from acute graft dysfunction to pyelonephritis, sepsis, and graft loss. Understanding the unique challenges UTIs present in renal transplant recipients is paramount for the healthcare community [2]. The epidemiology of UTIs in renal transplant recipients is particularly interesting. These infections are prevalent and often recurrent, contributing to a considerable burden of 4%-80% on healthcare resources [3]. Various factors, including post-transplant time frame, patient demographics, and comorbidities, influence the prevalence. Accurate data on the incidence and prevalence of UTIs in this population is essential for clinicians, researchers, and policymakers alike, as it guides the allocation of resources and the development of preventive strategies [3].

This comprehensive review aims to thoroughly examine UTIs in renal transplant recipients, mainly providing clinical insights and delineating effective management strategies. The specific objectives of this review encompass the following key areas: Firstly, to consolidate the current state of knowledge regarding the pathophysiology of UTIs in renal transplant recipients, highlighting the profound influence of immunosuppressive regimens, alterations in urinary tract anatomy, and microbial factors. Secondly, to explore the diverse clinical presentations of UTIs in this population, shedding light on atypical signs and symptoms, differential diagnostic considerations, and potential complications that may arise. Thirdly, to discuss the diagnostic approaches for UTIs, considering the unique challenges and intricacies associated with diagnosis in transplant recipients. Additionally, this review delves into the microbial origins of UTIs, encompassing not only common pathogens but also emerging threats and the far-reaching implications of antibiotic resistance. It further undertakes an in-depth analysis of management strategies, addressing critical aspects such as antibiotic selection, optimal treatment duration, prophylactic measures, and the pivotal role of antimicrobial stewardship.

## Review

Pathophysiology of UTIs in renal transplant recipients

Impact of Immunosuppression on UTI Risk

Immune deficiency: Immunosuppressive drugs used in the context of renal transplantation, which often include calcineurin inhibitors, antimetabolites, and corticosteroids, exert a profound influence on the recipient's immune system. These medications suppress the activity of various immune components, including T-cells, B-cells, and phagocytes. This extensive immune deficiency significantly impairs the recipient's ability to effectively defend against invading pathogens. Essentially, the immune system's capability to identify and combat microbial intruders is severely hampered, creating an environment conducive to infections [4].

Reduced barrier function: Immunosuppression goes beyond its effects on systemic immunity. It also compromises the natural defense mechanisms within the urinary tract. The mucosal lining, uroepithelium, and resident immune cells are essential in preventing pathogens from accessing the urinary tract. However, these protective barriers are weakened under the influence of immunosuppressive drugs. The uroepithelium, for instance, may become more permeable, allowing pathogens easier access to attach and invade the urinary tract. This compromised barrier function further amplifies the recipient's vulnerability to UTIs [5].

Latent infections reactivation: Immunosuppression weakens the immune system's defense against new infections and can also lead to the reactivation of latent infections. Many individuals harbor dormant viruses like cytomegalovirus (CMV) or Epstein-Barr (EBV). When the immune system is intentionally suppressed, these latent infections can reactivate. This reactivation diverts the immune system's attention from potential UTI-causing pathogens and directly impairs the host's immune response. As a result, the recipient becomes more susceptible to UTIs, as the immune system is preoccupied with controlling the reactivated viruses, leaving it less capable of dealing with other microbial threats in the urinary tract [6].

Altered Urinary Tract Anatomy and Function Post-transplant

Ureteral reimplantation: Ureteral reimplantation, a critical step in renal transplantation, involves connecting the donor kidney's ureter to the recipient's bladder. However, this surgical procedure may inadvertently result in anastomotic strictures or kinks within the ureter, potentially obstructing the smooth urine flow. When urine cannot pass freely, it can lead to urinary stasis, a well-established risk factor for UTIs. The stagnant urine creates an environment where bacteria can proliferate, increasing the likelihood of infection [7].

Vesicoureteral reflux: Vesicoureteral reflux is when urine flows backward from the bladder into the ureters. This occurrence can be observed in renal transplant recipients and poses a significant risk. When reflux transpires, it facilitates the ascent of bacteria from the bladder into the transplanted kidney. This retrograde flow of contaminated urine can introduce infectious agents into the renal pelvis, initiating UTIs and potentially complicating the management of the transplant [8].

Lymphocele formation: Lymphocele formation in the perirenal space is a potential post-transplant complication. These lymphatic fluid collections can exert pressure on the surrounding structures, including the urinary tract. Consequently, the altered pressure dynamics can disrupt the normal urine drainage from the kidney and predispose the patient to UTIs. The compromised urine flow and the possible introduction of bacteria from the lymphocele can create an environment conducive to infection [9].

Nerve denervation: The transplantation process may inadvertently result in nerve denervation of the transplanted kidney. This loss of neural connections can disrupt the normal bladder dynamics and micturition reflexes, potentially leading to urinary retention and incomplete bladder emptying. Urinary retention increases the risk of UTIs and stresses the transplanted kidney more. The inability to empty the bladder can result in residual urine, providing a fertile ground for bacterial growth and UTIs [10].

Microbial Factors Contributing to UTIs

Bacterial pathogens: UTIs in transplant recipients are frequently caused by various bacterial pathogens. The most common culprits are Escherichia coli, Klebsiella spp., Enterococcus spp., and Pseudomonas aeruginosa. These bacteria may originate from various sources, including the recipient's endogenous flora, the healthcare environment, or even the transplanted kidney. Identifying the specific pathogen responsible for the UTI is crucial for targeted treatment [11].

Virulence factors: Several uropathogenic bacteria have developed virulence factors that enhance their ability to cause UTIs. These virulence factors include adhesins and fimbriae, which allow the bacteria to adhere tightly to uroepithelial cells, evade host immune defenses, and even form resilient biofilms within the urinary tract. The ability to adhere to uroepithelial cells and create biofilms enhances the bacteria's persistence within the urinary tract, making them more challenging to eradicate with antibiotics. Understanding the virulence mechanisms of these pathogens is pivotal in developing effective treatment strategies [12].

Antibiotic resistance: The emergence of antibiotic-resistant strains of uropathogens poses a significant challenge in managing UTIs in transplant recipients. Multidrug-resistant bacteria, often due to the overuse and misuse of antibiotics, can substantially limit the available treatment options. In such cases, the risk of treatment failure increases, and alternative antibiotics may need to be considered. Therefore, monitoring antibiotic susceptibility patterns and the judicious use of antibiotics are essential to mitigate the risk of antibiotic resistance and to ensure effective treatment of UTIs in this vulnerable patient population [13].

Clinical presentation

Signs and Symptoms of UTIs in Transplant Recipients

UTIs in transplant recipients often manifest with a constellation of symptoms. Dysuria, characterized by a painful or burning sensation during urination, is a common complaint among these individuals. Additionally, UTIs frequently provoke increased urination frequency and urgency, resulting in discomfort and frequent restroom visits. Hematuria, the presence of blood in the urine, can vary from microscopic hematuria detected only through urine analysis to gross hematuria, which is visible to the naked eye. Some patients may report suprapubic pain or discomfort in the lower abdomen [14]. In contrast, others may experience flank pain, typically on one side, which can signal involvement of the upper urinary tract, such as pyelonephritis. Systemic symptoms like fever and chills may occur in more severe UTIs, particularly those affecting the kidneys. Notably, in elderly or immunocompromised transplant recipients, UTIs can present with non-specific symptoms such as altered mental status, confusion, delirium, or a decline in cognitive function, underscoring the diverse clinical presentations of UTIs in this population [15].

Atypical Presentations and Complications

Asymptomatic bacteriuria: Asymptomatic bacteriuria is a notable consideration in transplant recipients, particularly older people. This condition is characterized by bacteria in the urine without the typical symptoms of a UTI. Determining when to treat asymptomatic bacteriuria is a clinical challenge, as it may only sometimes necessitate intervention. The treatment decision should consider various factors, including the patient's age, underlying medical conditions, and the type of bacteria present. In elderly or immunocompromised individuals, treating asymptomatic bacteriuria is not always beneficial and may even contribute to antibiotic resistance [16].

Recurrent UTIs: Recurrent UTIs in transplant recipients can be vexing. They may result from various factors, including unresolved prior infections, anatomical abnormalities in the urinary tract, or complications arising from urological procedures post-transplant. These recurrent infections pose a significant clinical concern as they can negatively impact the patient's quality of life and potentially lead to complications such as graft dysfunction [17].

Pyelonephritis: UTIs can potentially progress to pyelonephritis, a severe kidney infection. Pyelonephritis is characterized by fever, flank pain, and systemic manifestations like chills. Pyelonephritis can lead to graft dysfunction and sepsis, a life-threatening condition when left untreated. Therefore, timely diagnosis and treatment of pyelonephritis in transplant recipients are crucial to prevent complications and preserve graft function [15].

Graft dysfunction: UTIs can result in acute graft dysfunction, often evidenced by elevated serum creatinine levels. However, distinguishing between graft rejection and graft dysfunction induced by a UTI can be a complex diagnostic challenge. Proper evaluation and careful clinical judgment are required to accurately identify the cause of graft dysfunction and initiate the appropriate treatment. This differentiation is vital as the treatment approaches for rejection and UTI-induced graft dysfunction differ significantly [18].

Differential Diagnosis Considerations

Graft rejection: Distinguishing between UTIs and acute graft rejection is a critical diagnostic challenge in renal transplant recipients. Acute graft rejection may manifest with elevated serum creatinine levels, which can also be observed in UTIs. This symptom overlap underscores the necessity of accurate differentiation between the two conditions. Rejection typically necessitates adjustments in immunosuppressive therapy, while UTIs require antimicrobial treatment [19].

Renal calculi: Kidney stones, or renal calculi, can give rise to symptoms that overlap with those of UTIs. Hematuria and flank pain, for instance, are commonly associated with both conditions. Accurate diagnosis is pivotal, as the management and treatment approaches for kidney stones and UTIs are distinct. Kidney stones may require procedures for removal or fragmentation, whereas UTIs demand appropriate antibiotic therapy [20].

Graft infections: Infections involving the transplanted kidney, such as abscesses or vascular infections, can present with clinical features resembling UTIs. Fever, flank pain, and even urinary symptoms can create diagnostic challenges. Distinguishing between graft infections and UTIs is crucial, as the management and therapeutic strategies differ significantly. Graft infections often necessitate surgical or interventional procedures in addition to antimicrobial therapy [2].

Medication side effects: Some immunosuppressive drugs administered to transplant recipients may induce side effects that can be misinterpreted as UTI symptoms. These side effects might include gastrointestinal symptoms like nausea, vomiting, diarrhea, and myelosuppression, which can manifest as fatigue and susceptibility to infections. To ensure appropriate management, healthcare providers must recognize and differentiate between these side effects and genuine UTIs [21].

Gynecological and urological conditions: Gynecological or urological conditions, such as cystitis or interstitial cystitis, can present urinary symptoms resembling UTIs. Patients with these conditions may experience dysuria, urinary frequency, and urgency, common UTI symptoms. Accurate diagnosis is necessary to provide patients with the appropriate treatment, which may differ from managing UTIs. These conditions often require specific therapeutic approaches, such as medications or lifestyle modifications [22].

Diagnostic approaches

Urinalysis and Urine Culture

Urinalysis and urine culture are pivotal diagnostic tools for evaluating renal transplant recipients' UTIs. Urinalysis encompasses physical and chemical urine assessments, including color, clarity, specific gravity, and critical markers like blood, protein, or nitrites. Additionally, microscopic examination aids in detecting white blood cells (pyuria) and red blood cells, which can signify infection or inflammation. Notably, leukocyte esterase and nitrites on dipstick testing hint at the possibility of a bacterial UTI [23]. On the other hand, urine culture is vital in pinpointing the responsible microorganism and determining its antibiotic susceptibility. This involves the collection of a midstream clean-catch urine sample, allowing the growth and identification of pathogenic bacteria [24].

Furthermore, quantitative urine culture results, expressed as colony-forming units per milliliter (CFU/mL), provide crucial insights for distinguishing between contamination and genuine infection. Antibiotic susceptibility testing assesses the effectiveness of various antibiotic options against the isolated pathogen to guide the selection of appropriate antibiotics. These diagnostic procedures are fundamental in ensuring accurate diagnosis and tailored treatment for UTIs in this medically complex patient population [25].

Imaging Studies

Renal ultrasound: Renal ultrasound is a non-invasive imaging technique that facilitates the evaluation of the transplanted kidney, ureters, and bladder. It is beneficial for identifying structural abnormalities like hydronephrosis, kidney stones, or abscesses. In addition, Doppler ultrasound can be utilized to assess renal blood flow and detect potential vascular complications, offering valuable information for diagnostic and treatment decisions [26].

CT scan: CT scans offer detailed cross-sectional images of the urinary tract, providing a comprehensive view of renal structures. They effectively identify renal abscesses, lymphoceles, and complications such as perinephric collections. Contrast-enhanced CT scans can reveal renal perfusion abnormalities and detect vascular complications, aiding in diagnosing and managing UTIs and associated complications [27].

Nuclear medicine scans: Nuclear medicine scans, including technetium-99m dimercaptosuccinic acid (DMSA) and diethylene triamine penta-acetic acid (DTPA) scans, offer valuable insights into renal function and parenchymal abnormalities. These scans play a pivotal role in assessing renal health and functionality, which is especially crucial in transplant recipients, as it aids in diagnosing and monitoring UTIs and their effects on the transplanted kidney [28].

Molecular Diagnostics and Biomarkers

Polymerase chain reaction (PCR): PCR is a molecular diagnostic technique with significant utility in detecting UTIs in renal transplant recipients. This method enables the sensitive and specific identification of the DNA of specific pathogens in urine samples, even at low concentrations. PCR assays hold value in identifying uropathogens that might not readily grow in standard laboratory settings, such as Mycoplasma or Ureaplasma species. These assays offer a valuable tool for precise pathogen identification, facilitating tailored treatment approaches and contributing to effective UTI management in this population [29].

Biomarkers: Biomarkers play an increasingly important role in diagnosing and assessing UTIs in transplant recipients. Established markers such as C-reactive protein (CRP), procalcitonin, and interleukin-6 (IL-6) indicate infection presence and severity, aiding clinical decision-making. Additionally, emerging urinary biomarkers like neutrophil gelatinase-associated lipocalin (NGAL) and kidney injury molecule-1 (KIM-1) are actively investigated for their potential in diagnosing and monitoring UTIs in transplant recipients. These biomarkers offer promising insights into renal health and function, allowing for early detection and monitoring of UTIs and ultimately improving patient care and outcomes [30].

Antibiotic Susceptibility Testing

Microbiological testing: Microbiological testing involves isolating and identifying the causative pathogens from urine cultures. These isolates are then subjected to antibiotic susceptibility testing, which assesses the sensitivity of the pathogen to various antibiotics. The results of these tests are pivotal in guiding the selection of antibiotics for treatment. They inform the choice of empirical therapy, providing a starting point for initial treatment before specific pathogen identification is available. Furthermore, they guide targeted antibiotic therapy, allowing healthcare providers to select the most effective antibiotics based on the identified pathogen's susceptibility profile. This tailored approach optimizes treatment outcomes and helps minimize the development of antibiotic resistance [31].

Microbial etiology

Common Pathogens Causing UTIs in Transplant Recipients

E. coli: E. coli is one of the most prevalent causative agents of UTIs in renal transplant recipients. Its widespread presence is attributed to its versatility and the various strains it encompasses. Certain strains of E. coli possess distinct virulence factors, which contribute to their pathogenicity. These factors can enhance their ability to adhere to uroepithelial cells, resist host immune defenses, and persist in the urinary tract, making E. coli a formidable uropathogen in this population [32].

Klebsiella species: Klebsiella species, particularly Klebsiella pneumoniae, are recognized as significant uropathogens in renal transplant recipients. What elevates their concern is their potential for multidrug resistance. This resistance can limit treatment options and complicate the management of UTIs, emphasizing the importance of selecting appropriate antibiotics based on susceptibility testing [33].

Enterococcus species: Enterococci, such as Enterococcus faecalis and Enterococcus faecium, are notable culprits behind UTIs, especially in transplants. They are often associated with resistance to multiple antibiotics, which poses a challenge in finding effective treatment options for UTIs caused by these pathogens [34].

P. aeruginosa: P. aeruginosa is a formidable threat in UTIs, as it can potentially induce severe infections. Of particular concern is its propensity to develop resistance to various antimicrobial agents, including some of the more potent antibiotics, making its management challenging and requiring specialized approaches [35].

Staphylococcus species: Although not as common as some previously mentioned pathogens, Staphylococcus species, such as Staphylococcus aureus or coagulase-negative staphylococci, can also lead to UTIs in renal transplant recipients. These infections may occur in catheter-related infections, underscoring the importance of vigilant catheter care and infection prevention in this patient population [36].

Proteus species: Proteus mirabilis is associated with complicated UTIs in renal transplant recipients. Its distinctive characteristics, including swarming motility and urease production, can lead to challenging and recurrent infections. Effective management strategies are essential to address Proteus-related UTIs in this population [37].

Emerging Pathogens and Antibiotic Resistance

Extended-spectrum beta-lactamase (ESBL)-producing bacteria: Enterobacteriaceae that produce Extended-Spectrum Beta-Lactamases, including certain strains of E. coli and Klebsiella, are increasingly recognized as significant contributors to UTIs in renal transplant recipients. These enzymes resist most beta-lactam antibiotics, rendering traditional treatment options less effective. The emergence of ESBL-producing bacteria necessitates carefully selecting antibiotics for UTI management in this population [38].

Carbapenem-resistant organisms: The rise of carbapenem-resistant Enterobacteriaceae (CRE) and carbapenem-resistant P. aeruginosa presents a growing concern for renal transplant recipients with UTIs. These organisms are particularly worrisome due to their resistance to carbapenem antibiotics, often considered last-resort treatments. Their limited antibiotic susceptibility underscores the need for alternative treatment strategies and heightened infection control measures [39].

Methicillin-resistant S. aureus (MRSA): MRSA is a formidable pathogen that can lead to UTIs, especially in healthcare-associated settings. MRSA strains often resist numerous antibiotics, making them challenging to treat. In the context of renal transplant recipients, alternative therapeutic approaches become essential to address MRSA-related UTIs effectively [40].

Aminoglycoside-resistant bacteria: Certain uropathogens have developed resistance to aminoglycoside antibiotics, significantly limiting the utility of these drugs for UTI treatment. The reduced efficacy of aminoglycosides underscores the importance of carefully selecting antibiotics based on susceptibility testing, especially when treating UTIs in transplant recipients [41].

Multidrug-resistant gram-negative bacteria: Multidrug-resistant Gram-negative bacteria, including but not limited to Acinetobacter baumannii, pose a significant concern for renal transplant recipients and can lead to complicated UTIs. These organisms have developed resistance to multiple antibiotics, necessitating specialized and often combination therapies to manage UTIs in this vulnerable patient population effectively [42].

Viral and Fungal UTIs in This Population

Cytomegalovirus (CMV): CMV is a well-recognized viral pathogen that can give rise to viral UTIs in transplant recipients, including renal transplant recipients. These infections may be prevalent when the transplant recipient is CMV-seronegative and receives an organ from a CMV-seropositive donor. In such cases, the risk of CMV transmission is heightened. CMV-related UTIs can pose significant challenges, as the virus can cause various urinary symptoms and complications, including urethritis and cystitis. The management typically involves antiviral therapy and may require close monitoring to prevent the progression of CMV infection [43].

Adenovirus: Adenovirus infections have the potential to lead to hemorrhagic cystitis, a viral UTI characterized by hematuria (blood in the urine) and inflammation of the bladder. This condition can manifest in renal transplant recipients and is often associated with using immunosuppressive medications. Adenovirus-induced UTIs, particularly hemorrhagic cystitis, can be painful and may necessitate supportive care, including hydration and pain management. In severe cases, antiviral treatment may be considered [44].

Fungal UTIs: Fungal UTIs, particularly candiduria due to Candida species, can occur in transplant recipients, particularly when broad-spectrum antibiotics have been used. These antibiotics can disrupt the average balance of microorganisms in the urinary tract, creating an environment conducive to fungal overgrowth. Candiduria can lead to symptoms such as dysuria, frequency, and urgency. Treatment involves antifungal therapy, often tailored to the Candida species identified in urine cultures. Additionally, addressing any underlying risk factors, such as the judicious use of antibiotics and improved infection control, is essential to prevent fungal UTIs in transplant recipients [45].

Management strategies

Antibiotic Selection and Dosing Considerations

Empirical antibiotic therapy: Antibiotic therapy is a crucial initial step in managing renal transplant recipients' UTIs. This approach involves promptly initiating antibiotic treatment while awaiting urine culture results. Common empirical choices include antibiotics like ciprofloxacin, levofloxacin, or ceftriaxone, which provide coverage against a broad spectrum of uropathogens. However, it's essential to recognize that the choice of empirical antibiotics should be informed by local resistance patterns and the patient's specific needs, as resistance can vary by region and patient population. Tailoring empirical therapy to local resistance profiles helps maximize its effectiveness [18].

Antibiotic susceptibility testing: The treatment plan should be adjusted based on the identified pathogen's susceptibility profile upon receiving urine culture results. This step ensures targeted therapy by selecting antibiotics to which the causative pathogen is sensitive. By using this information, healthcare providers can optimize the treatment regimen, enhancing the likelihood of successful resolution of the UTI and minimizing the risk of antibiotic resistance development [25].

Dosing adjustments: Renal transplant recipients often require dosing adjustments for antibiotics due to the altered renal function resulting from transplantation and immunosuppressive medications. These dosing modifications should be guided by parameters like creatinine clearance or estimated glomerular filtration rate (eGFR). They ensure antibiotics are dosed appropriately to avoid suboptimal treatment or potential toxicity [46].

Avoid nephrotoxic agents: Renal transplant recipients are particularly susceptible to nephrotoxicity, and certain antibiotics, such as aminoglycosides and vancomycin, are known to be nephrotoxic. It is crucial to exercise caution and avoid these agents when possible to prevent further compromise of graft function. To safeguard renal health, healthcare providers should prioritize antibiotics with lower nephrotoxic potential in this patient population [47].

Duration of Treatment

Uncomplicated UTIs: Uncomplicated lower UTIs are generally treated with shorter courses of antibiotics, often as brief as three days, provided the selected antibiotic is effective against the causative pathogen, and the patient responds favorably. The relatively short treatment duration is appropriate for infections localized to the lower urinary tract and is less likely to involve deeper tissues or have complications. The goal is to eradicate the infection while minimizing the risk of antibiotic resistance and adverse effects associated with longer courses [48].

Complicated UTIs: Complicated UTIs, which may include conditions like pyelonephritis or those associated with anatomical abnormalities, often demand a longer course of treatment, typically seven to 14 days. These infections are more likely to extend beyond the lower urinary tract, potentially involving deeper tissues, and carry a higher risk of complications. A prolonged treatment duration is required to ensure that the infection is effectively eradicated and to prevent recurrence or the development of more severe complications [48].

Recurrent UTIs: Recurrent UTIs can be particularly challenging, especially in renal transplant recipients. In these cases, healthcare providers may consider prophylactic or suppressive antibiotic therapy. This approach involves providing patients with low-dose antibiotics over an extended period, typically based on the patient's clinical history and susceptibility patterns. Prophylactic therapy aims to prevent or reduce the frequency of recurrent UTIs in patients at higher risk due to underlying factors such as anatomical abnormalities or frequent infections [17].

Prophylactic Strategies

Pre-transplant evaluation: A comprehensive pre-transplant assessment of potential recipients is crucial in preventing post-transplant UTIs. This evaluation helps identify and treat pre-existing UTIs and urological issues in prospective recipients. Healthcare providers can reduce the risk of introducing infections into the transplant setting by addressing these concerns before transplantation, thus promoting better post-transplant outcomes [49].

Immunosuppression optimization: The careful optimization of immunosuppressive regimens is essential in the post-transplant period. By tailoring these regimens to balance the prevention of graft rejection with the risk of UTIs, healthcare providers can minimize the likelihood of post-transplant UTIs while preserving graft function. Achieving this balance is critical for the long-term success of the transplant [50].

Catheter management: Minimizing the use of urinary catheters and employing aseptic techniques during catheter insertion and care are critical strategies for reducing the risk of catheter-associated UTIs. Catheters should only be used when necessary, and their removal should be expedited to limit the duration of catheterization. In cases where catheters are essential, strict adherence to infection prevention protocols is vital to prevent UTIs [51].

Hygiene and lifestyle: Patient education on proper hygiene practices, staying well-hydrated, and avoiding urinary tract irritants is fundamental in preventing UTIs in renal transplant recipients. Proper hygiene includes teaching patients the importance of regular handwashing and perineal care. Encouraging individuals to maintain good hydration and avoid known urinary irritants, such as excessive caffeine or alcohol, can help create a less favorable environment for developing UTIs [2].

Management of Complicated UTIs

Urological assessment: A thorough urological assessment is paramount in renal transplant recipients' UTIs. This evaluation involves using imaging techniques and consultation with urology specialists to identify and address any anatomical abnormalities or obstructive conditions that may contribute to the UTI. By detecting and addressing these issues, healthcare providers can reduce the risk of recurrent infections and complications that could impact the transplanted kidney's health and function [48].

Antibiotic therapy: The timely initiation of effective and targeted antibiotic therapy is a cornerstone of UTI management in renal transplant recipients. Initial treatment is often administered intravenously to ensure rapid and reliable drug delivery. However, the choice of antibiotics may be modified based on the results of urine culture and sensitivity testing, which helps identify the specific pathogen and its susceptibility profile. Adapting treatment to the causative organism's susceptibility pattern ensures that the selected antibiotics are most effective in eradicating the infection [52].

Hospitalization: Severe complicated UTIs may necessitate hospitalization, particularly those involving pyelonephritis. Hospitalization provides an opportunity for intensive treatment with intravenous antibiotics and fluid resuscitation, which is crucial for managing severe infections. Close monitoring of graft function is also essential during hospitalization to ensure that the transplanted kidney remains healthy and unaffected by the infection or its treatment. Inpatient care allows for close observation and therapy adjustment to optimize outcomes [53].

Surgical intervention: In some instances of UTIs in renal transplant recipients, surgical intervention may be required. This is especially true for situations involving the formation of abscesses, obstructive uropathies, or persistent complications that do not respond adequately to antibiotic therapy alone. Surgical procedures may be needed to drain abscesses, correct anatomical abnormalities, or alleviate obstructions to resolve the underlying issues and prevent further infections [48].

Role of Antimicrobial Stewardship

Regular review: Ensuring the judicious use of antibiotics in managing UTIs in renal transplant recipients necessitates ongoing and systematic review by a multidisciplinary team. This team typically includes infectious disease specialists and clinical pharmacists who work collaboratively to monitor and adjust antibiotic regimens. This review process should be based on the results of urine cultures and the guiding principles of antimicrobial stewardship. By regularly assessing the effectiveness of antibiotics and considering changes as necessary, the healthcare team can optimize treatment outcomes while minimizing the development of antibiotic resistance [54].

De-escalation: When deemed appropriate, antibiotics used to treat UTIs should be de-escalated to narrower-spectrum agents. De-escalation involves transitioning from broad-spectrum antibiotics to more targeted options. This strategy helps minimize collateral damage to the normal flora within the urinary tract and reduces the risk of antibiotic resistance. By tailoring the antibiotic regimen to target the identified pathogen specifically, healthcare providers can maintain the delicate balance between effective treatment and preserving the microbial ecosystem within the urinary tract [55].

Education: Education is a pivotal component of antimicrobial stewardship in treating UTIs in renal transplant recipients. Healthcare providers, including transplant recipients themselves, should be well-informed about the principles of antimicrobial stewardship, rational antibiotic use, and the importance of completing prescribed courses of antibiotics. Education empowers patients and providers to make informed decisions regarding antibiotic therapy, which, in turn, contributes to more effective and responsible antibiotic use. Patient education plays a crucial role in enhancing adherence to treatment regimens and minimizing the development of resistance [56].

Prevention and risk reduction

Pre-transplant Patient Evaluation

Screening for pre-existing UTIs: Thoroughly screening renal transplant recipients for pre-existing UTIs is essential in the pre-transplant evaluation process. Identifying and promptly treating any existing UTIs is crucial for several reasons. First, untreated infections can lead to complications during and after transplantation. Second, they may increase the risk of infecting the transplanted kidney during the surgical procedure. By addressing pre-existing UTIs before transplantation, healthcare providers can reduce the likelihood of complications and help safeguard both the patient and the transplanted organ [54].

Urological assessment: A comprehensive urological assessment is vital to detect anatomical abnormalities in the pre-transplant phase of the urinary tract. If identified, conditions such as urethral strictures or vesicoureteral reflux can be corrected before transplantation. Correcting these abnormalities reduces the risk of UTIs and other complications post-transplant, promoting the long-term success of the graft [57].

Assessment of comorbidities: An essential aspect of the pre-transplant evaluation involves evaluating the recipient's medical history for comorbidities that may increase the risk of UTIs. Conditions like diabetes, which can compromise the immune system and predispose individuals to infections, should be optimized and managed effectively before transplantation. By controlling comorbidities, healthcare providers can mitigate the risk of post-transplant UTIs and enhance the recipient's overall health [48].

Immunosuppression risk assessment: Choosing an immunosuppressive regimen is a critical consideration in the pre-transplant phase, as it can influence the risk of UTIs. Healthcare providers should assess the immunosuppression regimen's risk profile regarding UTIs and tailor it to minimize this risk while ensuring graft function and minimizing the risk of graft rejection. Achieving this balance is essential for the recipient's post-transplant health and the success of the transplant itself [54].

Perioperative Strategies

Aseptic techniques: During the renal transplant procedure, the use of aseptic techniques is of paramount importance to prevent contamination of the graft, urinary tract, and surgical site. These techniques include rigorous hand hygiene, sterilization of surgical instruments, and maintaining a sterile field in the operating room. Adhering to aseptic principles, healthcare providers can minimize the risk of introducing infections during the transplantation, which is crucial for the procedure's success and the recipient's long-term health [58].

Ureteral reimplantation: Ureteral reimplantation, which involves connecting the donor kidney's ureter to the recipient's bladder, demands meticulous surgical techniques. Careful attention to detail is essential to reduce the risk of ureteral strictures or obstructions, leading to urinary stasis and increasing the risk of UTIs. The surgical team should ensure that the ureter is anastomosed without tension and with minimal risk of complications, thus preserving the integrity of the urinary tract [59].

Prophylactic antibiotics: The perioperative administration of prophylactic antibiotics is a well-established practice to reduce the risk of immediate post-transplant UTIs. Antibiotic selection for prophylaxis should consider local resistance patterns to ensure the chosen agents are effective against the most likely pathogens. Prophylactic antibiotics help prevent infections that could compromise the early success of the transplant and reduce the need for post-transplant treatment, thus improving patient outcomes [60].

Catheter care: Minimizing the use of urinary catheters and implementing sterile catheter insertion and maintenance procedures are vital strategies to reduce the risk of catheter-associated UTIs. Urinary catheters should only be used when clinically necessary, and their duration should be limited to prevent potential pathogens' colonization of the urinary tract. Adhering to strict infection prevention protocols during catheter insertion and care further reduces the risk of UTIs associated with catheter use [61].

Post-transplant Follow-Up and Monitoring

Regular surveillance: Implementing a schedule for regular follow-up visits and urinalysis is crucial for monitoring renal transplant recipients. These surveillance measures are designed to detect signs of infection, such as hematuria or leukocyturia, at an early stage. Regular follow-up ensures that any potential UTIs are identified and treated promptly, thus reducing the risk of complications and graft-related issues [54].

Renal function monitoring: Continuous monitoring of graft function is essential to post-transplant care. This involves frequent assessments of serum creatinine levels and estimated glomerular filtration rate (eGFR). Scrutiny of renal function allows healthcare providers to promptly identify any anomalies indicative of UTIs or other complications. By maintaining optimal graft function, recipients are better equipped to resist infections and achieve successful long-term outcomes [62].

Immunosuppressive adjustments: The tailoring of immunosuppressive regimens is integral to post-transplant management. Adjustments to these regimens should be based on graft function and the risk of UTIs. The goal is to strike a delicate balance between minimizing the risk of infection and protecting the transplanted graft from rejection. Careful consideration of immunosuppression allows healthcare providers to adapt treatment plans as needed, ensuring recipients receive the most appropriate care [63].

Education: Educating transplant recipients on critical aspects of post-transplant care is essential. This education should cover the importance of proper hygiene, adequate fluid intake, and recognizing the signs and symptoms of UTIs. Equipping recipients with this knowledge empowers them to actively participate in their health and well-being. Early recognition of UTIs and timely reporting of symptoms to healthcare providers facilitate prompt intervention and the prevention of complications [64].

Vaccination Considerations

Influenza and pneumococcal vaccination: Encouraging the vaccination of renal transplant recipients against influenza and pneumococcal infections is paramount. Influenza and pneumococcal diseases can exacerbate the risk of UTIs by causing systemic illnesses that impair immune function. By vaccinating transplant recipients against these pathogens, healthcare providers can reduce the likelihood of systemic infections that may predispose the patient to UTIs and other complications [65].

Viral vaccination: Consideration should be given to vaccination against specific viruses, such as hepatitis B, hepatitis C, and CMV. These vaccinations may indirectly reduce the risk of UTIs by preventing systemic viral infections that can affect the urinary tract. Preventing these viral infections can help maintain the overall health and immunity of the transplant recipient, lowering the susceptibility to UTIs [66].

Vaccination in seronegative recipients: In cases where renal transplant recipients are CMV-negative and receive an organ from a CMV-positive donor, post-transplant prophylaxis or pre-emptive therapy may be considered to prevent CMV infection. CMV infections can lead to viral UTIs and other complications. The choice of prophylaxis or therapy should be guided by clinical guidelines and tailored to the specific risk profile of the recipient [43].

Vaccination and immunomodulatory therapies: It is crucial to ensure that vaccination decisions consider the immunosuppressive regimens that transplant recipients are prescribed. Some medications used in immunosuppressive therapy may interfere with the patient's ability to develop protective immune responses to vaccines. Healthcare providers should carefully assess the individual patient's situation, considering both the need for vaccination and the potential impact of immunosuppressive medications on vaccine efficacy [67].

Complications and outcomes

Impact of UTIs on Graft and Patient Survival

Graft dysfunction: One of the primary concerns associated with UTIs in renal transplant recipients is the potential for acute graft dysfunction. UTIs can lead to inflammation and infection within the transplanted kidney, impairing graft function. This dysfunction is often reflected in elevated serum creatinine levels, a key marker of renal function. Prompt diagnosis and treatment of UTIs are crucial to prevent long-term damage to the graft. Unresolved or severe UTIs can exacerbate graft dysfunction and compromise the recipient's health [1].

Allograft loss: Severe or recurrent UTIs can contribute to allograft loss, especially if they lead to irreversible damage to the transplanted kidney. The transplanted organ is a precious resource, and any condition jeopardizing its function or viability is a great concern. UTIs that cause significant harm to the graft may ultimately result in allograft loss, necessitating additional transplant interventions [68].

Patient survival: The impact of UTIs on patient survival is multifaceted. Severe UTIs can lead to systemic infection and sepsis, significantly affecting patient outcomes. In cases where UTIs progress to more severe infections, the mortality risk is elevated. Timely and effective management of UTIs is essential for preserving graft function and ensuring the survival and well-being of renal transplant recipients [69].

Recurrence and chronic graft dysfunction: Repeated UTIs may contribute to chronic graft dysfunction. The persistent inflammation and damage caused by recurrent infections can gradually lead to allograft failure. Chronic graft dysfunction is a severe complication that can affect the long-term health of transplant recipients. Preventing UTI recurrence and managing these infections effectively is essential to mitigate the risk of chronic graft dysfunction [48].

Rejection Risk and Immunosuppressive Adjustments

Rejection risk: UTIs can significantly impact the risk of graft rejection in renal transplant recipients. UTIs trigger an inflammatory response in the urinary tract and can influence the recipient's immune status. This inflammation and activation of the immune system can increase the risk of acute or chronic graft rejection. Graft rejection occurs when the recipient's immune system identifies the transplanted organ as foreign and mounts an immune response against it. UTIs may exacerbate this immune response and contribute to graft rejection, highlighting the need for prompt diagnosis and treatment of UTIs to minimize this risk [1].

Immunosuppressive adjustments: To manage the delicate balance between the risk of graft rejection and the risk of infection, healthcare providers may need to modify the immunosuppressive regimen in some cases. During active UTIs, reducing the level of immunosuppression may be necessary to allow the recipient's immune system to mount a more effective defense against the infection. However, these adjustments must be cautiously made, as lowering immunosuppression too much can increase the risk of graft rejection. Balancing these competing risks requires careful clinical judgment and individualized treatment decisions [70].

Therapeutic drug monitoring: Close monitoring of immunosuppressive drug levels is crucial in the post-transplant care of renal transplant recipients. This monitoring helps ensure the patient receives adequate immunosuppression to prevent graft rejection while minimizing the risk of infections, including UTIs. Therapeutic drug monitoring involves regular assessments of drug levels in the patient's blood and adjustments to the immunosuppressive regimen as needed. This practice is a critical component of transplant management, as it allows healthcare providers to fine-tune the patient's treatment to maintain a delicate equilibrium between immune suppression and protection [71].

Long-Term Consequences of Recurrent UTIs

Chronic kidney disease: Recurrent UTIs can pose a substantial risk to the renal transplant recipient by contributing to chronic kidney disease (CKD). These repeated infections may lead to persistent inflammation and damage to the transplanted kidney, resulting in a progressive decline in graft function. CKD is a serious long-term consequence of recurrent UTIs, highlighting the critical need to address and manage these infections effectively to protect the patient's renal health [72].

Antibiotic resistance: Frequent antibiotic use to treat recurrent UTIs can inadvertently promote the development of antibiotic-resistant uropathogens. The overuse or misuse of antibiotics can select strains of bacteria that are less susceptible to these drugs, making future UTIs harder to treat. Antibiotic resistance is a growing global concern, and its implications for transplant recipients are especially significant. This underscores the importance of judicious antibiotic use and a thoughtful approach to UTI management to mitigate the risk of resistance [73].

Interventions: Healthcare providers may need to consider interventions beyond standard antibiotic treatment in frequent or severe recurrent UTIs. Urological procedures, such as addressing anatomical abnormalities or ureteral strictures, may be necessary to reduce the risk of recurrent infections. Additionally, suppressive antibiotic therapy, which involves the long-term use of low-dose antibiotics, may be considered to prevent or minimize UTI recurrence. These interventions should be tailored to the patient's circumstances and risk factors [48].

Quality of life: Recurrent UTIs can profoundly impact the patient's quality of life. These infections' psychological and physical toll and the need for frequent medical interventions can significantly affect the recipient's overall well-being. Effective preventive strategies, close monitoring, and proactive management of UTIs are essential to reduce the burden on patients and help them maintain a high quality of life post-transplant [74].

Future directions and research needs

Potential Therapies and Preventative Measures

Antibacterial prophylaxis: The development of targeted antibacterial prophylactic strategies, guided by individual susceptibility and microbiome analysis, holds promise for reducing the risk of UTIs in renal transplant recipients. Tailoring prophylaxis to the patient's specific microbial profile and susceptibility factors can help prevent UTIs more effectively. Personalized antibacterial prophylaxis aims to protect uropathogens most likely to cause infections in individual recipients, improving the overall management of UTI risk [75].

Vaccine development: Research into vaccines targeting common uropathogens, particularly in the context of renal transplantation, offers a novel approach to preventing UTIs. Vaccines can stimulate the recipient's immune system to recognize and defend against specific pathogens, reducing the likelihood of infection. Developing effective and safe vaccines for transplant recipients could significantly contribute to UTI prevention, reducing reliance on antibiotics and minimizing the risk of antibiotic resistance [76].

Microbiome modulation: Investigating interventions to modulate the urinary and gut microbiome to promote a balanced microbial environment is an exciting avenue for reducing the risk of infection. Modifying the microbiome to enhance its protective functions and discourage the growth of uropathogens may be a future strategy in UTI prevention. Approaches could include probiotics, prebiotics, or other therapies that promote a healthy microbial balance and bolster the body's natural defenses against infections [77].

Host-targeted therapies: Developing host-targeted therapies that enhance the urinary tract's innate immune defenses represents an innovative strategy for preventing UTIs. These therapies aim to strengthen the recipient's immune response and protective mechanisms in the urinary tract, making it more resistant to infections. By bolstering the host's ability to ward off uropathogens, these therapies offer an alternative to traditional antimicrobial approaches and may reduce the need for antibiotics in UTI prevention [78].

Areas for Further Study and Clinical Trials

Optimal immunosuppression: Research on determining the most effective immunosuppressive regimens that balance graft protection and infection risk reduction is critical. Optimizing immunosuppression is a complex challenge in the care of transplant recipients, and understanding the best approaches can lead to improved patient outcomes and reduced UTI risk [79].

Antibiotic resistance: Investigation into the prevalence and mechanisms of antibiotic resistance in uropathogens is essential, specifically focusing on strategies to combat resistance. Antibiotic-resistant infections pose a significant threat to transplant recipients, and research into strategies to address this challenge, such as novel antibiotics or alternative treatment approaches, is crucial [80].

Biomarkers: Identifying novel biomarkers for the early detection and monitoring of UTIs, including predictive markers for high-risk patients, can significantly enhance the management of these infections. Biomarkers can enable timely intervention, reducing the severity and consequences of UTIs [81].

Patient-specific risk profiling: The development of personalized risk profiles that consider individual patient characteristics, medical history, and susceptibility to UTIs is a valuable avenue for research. Tailoring prevention and treatment strategies to each patient's needs can lead to more effective care and better outcomes [82].

Long-term outcomes: Investigating the long-term impact of UTIs on graft survival, patient quality of life, and healthcare resource utilization is critical for understanding the broader implications of these infections. This research can guide healthcare providers in developing comprehensive care plans and optimizing the post-transplant experience for renal transplant recipients [83].

Advances in Personalized Medicine for UTI Management

Genomic profiling: Genomic profiling is a cutting-edge approach to understanding renal transplant recipients' UTIs. By integrating genomic information, researchers can identify genetic predispositions that may make specific individuals more susceptible to UTIs. This personalized approach allows for tailoring preventive and therapeutic strategies based on an individual's genetic profile. For instance, identifying specific genetic markers associated with increased UTI risk could inform targeted interventions to reduce susceptibility and enhance patient outcomes [84].

Microbiome analysis: Microbiome analysis, focusing on the urinary and gut microbiota, is a burgeoning field with significant potential in UTI prevention and management. This approach involves studying the composition and diversity of microbial communities in these areas. By understanding the microbial balance, researchers can guide interventions that maintain a healthy microbiome, which, in turn, can help prevent UTIs. Strategies might include probiotics, prebiotics, or other microbial modulators to promote the growth of beneficial microorganisms and suppress uropathogens [85].

Immunosuppression tailoring: Customizing immunosuppressive regimens is crucial for optimizing the balance between graft protection and infection risk reduction. Immunosuppression tailoring involves tailoring these drug regimens based on individual immune profiles, infection risk factors, and the stability of the transplanted organ. By fine-tuning the immunosuppression strategy for each patient, healthcare providers can achieve a more precise and practical approach to managing UTI risk [70].

Antibiotic selection: The concept of precision medicine extends to antibiotic selection. Instead of a one-size-fits-all approach, precision medicine involves choosing the most effective antibiotics based on susceptibility testing and individual patient factors. This approach minimizes the overuse of broad-spectrum antibiotics and selects the most targeted treatment for the specific pathogen causing the UTI. Precision antibiotic selection can enhance the effectiveness of treatment, reduce collateral damage to the microbiome, and mitigate the risk of antibiotic resistance [86].

Risk prediction models: Developing predictive models for UTI risk is an interdisciplinary approach integrating clinical, immunological, and microbiological data. These models use a combination of patient-specific factors, such as medical history, immune status, and microbiome composition, to assess the likelihood of UTIs in transplant recipients. By predicting risk, healthcare providers can implement proactive measures, such as personalized preventive strategies, to reduce the incidence and severity of UTIs. Risk prediction models contribute to more targeted and effective care [87].

## Conclusions

In conclusion, managing UTIs in renal transplant recipients is a multifaceted challenge that demands a comprehensive approach. Key clinical insights highlight the elevated risk factors associated with transplant recipients, including immunosuppression, altered urinary tract anatomy, and complex comorbidities. The complications of UTIs, such as graft dysfunction, allograft loss, and systemic illness, underscore the significance of effective management. The emergence of multidrug-resistant uropathogens further complicates treatment, necessitating targeted antibiotic therapy. Preventive measures involving pre-transplant evaluation, perioperative strategies, post-transplant follow-up, and vaccination are essential for reducing the incidence of UTIs. Collaborative care from a multidisciplinary team, including transplant specialists, infectious disease experts, pharmacists, and patient education, is pivotal in successfully managing UTIs in renal transplant recipients. By applying these strategies and continuing to advance research in areas such as personalized medicine and novel therapies, healthcare providers can enhance patient care, leading to improved graft and patient survival in this vulnerable population.
